# The effect of volume equated 1- versus 2-day formats of Nordic hamstring exercise training on fitness in youth soccer players: A randomised controlled trial

**DOI:** 10.1371/journal.pone.0277437

**Published:** 2022-12-29

**Authors:** Jason Moran, Norodin Vali, Ben Drury, Raouf Hammami, Jamie Tallent, Helmi Chaabene, Rodrigo Ramirez-Campillo

**Affiliations:** 1 School of Sport, Rehabilitation, and Exercise Sciences, University of Essex, Colchester, United Kingdom; 2 Department of Exercise Physiology, Shahid Rajaee Teacher Training University, Tehran, Iran; 3 Department of Sport, Hartpury University, Gloucestershire, United Kingdom; 4 Higher Institute of Sport and Physical Education of Ksar-Said, Universite de La Manouba, Tunis, Tunisia; 5 Research Laboratory: Education, Motor Skills, Sports and Health, Higher Institute of Sport and Physical Education of Sfax, University of Sfax, Sfax, Tunisia; 6 Department of Sports and Health Sciences, Faculty of Human Sciences, University of Potsdam, Potsdam, Germany; 7 Higher Institute of Sports and Physical Education, Kef, University of Jendouba, Jendouba, Tunisia; 8 Exercise and Rehabilitation Sciences Laboratory, Faculty of Rehabilitation Sciences, School of Physical Therapy, Universidad Andres Bello, Santiago, Chile; Instituto Politecnico de Viana do Castelo, PORTUGAL

## Abstract

**Purpose:**

This randomised controlled trial examined the effect of an 8-week volume-equated programme of Nordic hamstring exercise (NHE) training, executed at frequencies of 1- or 2-days per week, on fitness (10 m and 40 m sprint, ‘505’ change of direction [COD] and standing long jump [SLJ]) in male youth soccer players (mean age: 16.4 ± 0.81 years).

**Method:**

Players were divided into an experimental group (n = 16) which was further subdivided into 1-day (n = 8) and 2-day (n = 8) per week training groups and a control group (n = 8).

**Results:**

There were significant group-by-time interactions for 10-m sprint (p<0.001, η^2^ = 0.120, d = 2.05 [0.57 to 3.53]), 40-m sprint (p = 0.001, η^2^ = 0.041, d = 1.09 [-0.23 to 2.4]) and COD (p = 0.002, η^2^ = 0.063, d = 1.25 [-0.09 to 2.59). The experimental group demonstrated a ‘very large’ effect size (d = 3.02 [1.5 to 4.54]) in 10-m sprint, and ‘large’ effect sizes in 40-m sprint (d = 1.94 [0.98 to 2.90]) and COD (d = 1.84 [0.85 to 2.83). The control group showed no significant changes. There were no significant differences between the 1-day and 2-day training groups. In three of the four tests (40 m, COD, SLJ) the 2-day group demonstrated larger effect sizes. Ratings of perceived exertion (RPE) were significantly lower in the 2-day group (p<0.001, 3.46 [1.83 to 5.04).

**Conclusion:**

The NHE increases fitness in youth soccer players and there may be advantages to spreading training over two days instead of one.

## Introduction

The Nordic Hamstring Exercise (NHE) has previously been shown to drive architectural changes that mediate strength increases in the hamstrings [1–5]. Indeed, eccentric hamstring strength increases of 15% have been observed in youth athletes over a short period of time (i.e. 5–6 weeks) [6, 7]. Similarly, recent research has demonstrated that the NHE can have positive effects on other important physical qualities such as sprint and change of direction (COD) speed in youth performers, even in the absence of architectural changes [8]. However, despite its efficacy, reservations about the use of the NHE in youth populations have been expressed due to its supposedly high level of intensity which could result in delayed onset muscle soreness [9]. Moreover, notwithstanding its efficacy, there is still a dearth of evidence on how the NHE can be optimally programmed in a youth athlete’s schedule, a particular concern in the time-constrained environment of modern soccer [10, 11]. This could lead to a reprioritisation of training activities, resulting in less time being devoted to strength and conditioning activities. Accordingly, methods that can ensure the maintenance of optimal training loads are imperative.

Most published research has seen the NHE prescribed with training frequencies of two or three times per week [12]. There has been comparatively little exploration of once per week protocols which could be of use to coaches who operate within time-constrained environments [11]. Medeiros et al. [11] investigated the effect of 1- and 2-day per week NHE frequencies on injury risk factors in adult soccer players, finding that only the 2-day protocol resulted in increased strength of the hamstrings. However, the 2-day group executed twice as many repetitions as the 1-day group meaning the effects *of volume-equated* resistance training (RT) of different training frequencies remains unclear. A meta-analysis by Grgic et al. [13] reported that greater RT volumes, resulted in enhanced muscular strength. However, when RT volumes were distributed evenly across different configurations of weekly training sessions, gains in strength occurred in a similar pattern (though this was not specifically in relation to NHE training). The authors indicated that additional studies on the effect of different RT frequencies were needed to draw more conclusive inferences. However, it is also interesting to note that of the 22 studies included in the aforementioned review [13], only one [14] was conducted in the young target population for our investigation. Similarly, a meta-analysis by Cuthbert et al. [15] revealed no significant differences in strength when well-trained populations were exposed to volume-equated RT programmes of different weekly frequencies. The authors concluded that RT could be structured in short, more frequent sessions that could be programmed around the competitive schedule but, again, none of the included studies were conducted in a youth population.

Contrary to the above evidence, Schoenfeld et al. [16] conducted a meta-analysis that indicated that volume-equated RT programs were more effective for increasing muscle hypertrophy when conducted over two days per week than they were over one. Muscle hypertrophy has been shown to be one of the primary drivers of strength increases in response to RT and is one of the main goals in programmes of physical preparation for sport [17, 18]. More recently, in an intervention conducted in young untrained males (age: 22.3 ± 0.9 years), Ochi et al. [19] demonstrated that three sessions per week (2 sets of 12 repetitions per session) of knee extension exercise was superior to one session per week (6 sets of 12 repetitions). With both groups carrying out 72 repetitions per week, Ochi et al. [19] reported significantly higher increases in strength in the group that executed these across three sessions, as opposed to one (65.2% vs. 43.5%). It is notable that ratings of perceived exertion (RPE) were higher in the one-session group than they were in the three-session group, suggesting that spreading the same amount of training across a higher number of sessions might minimise the negative effects of physical exertion on performance.

Previous work [11, 20] in this area has recommended that researchers should evaluate the extent to which NHE training programmes can affect physical fitness tests relating to jumping, sprinting and deceleration capability. These are important physical qualities in youth soccer as such dynamic movements have been shown to occur prior to the scoring of goals [21]. Previous work [6, 7] has also examined the various effects of the NHE on performance in youth athletes. However, to our knowledge, no study has evaluated the effect of volume-equated NHE loads, executed in different weekly configurations, on physical performance in this population. This may be an important factor for coaches who wish to maximise training adaptations in time-constrained environments [10, 11]. The purpose of this randomised controlled trial was to determine the effect of eight weeks of NHE training on physical performance in youth soccer players. A concurrent objective was to investigate the effect of a volume-equated programme of NHE, executed at frequencies of one or two training sessions per week, on physical performance. We hypothesised that the applied NHE programme would elicit significant increases in physical performance and that improvements would be of a similar magnitude whether performing the NHE either once or twice per week.

## Materials and methods

The study was conducted according to the latest version of the Declaration of Helsinki and the protocol was approved by the University of Essex ethics committee prior to the commencement of any assessments. Participants and their parents gave their written consent to partake in the study.

### Participants

**Fig 1:**
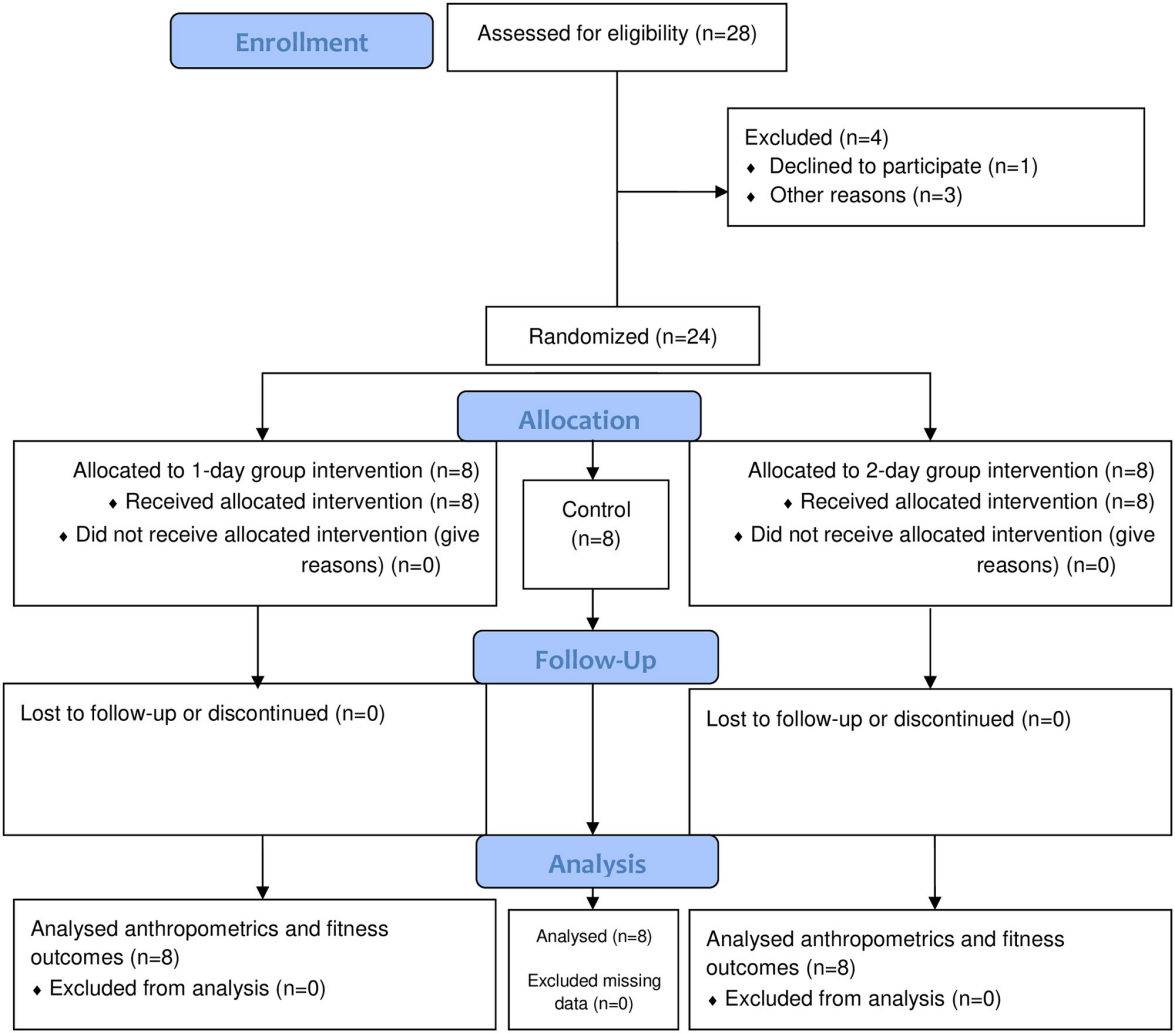
CONSORT (Consolidated Standards of Reporting Trials) diagram.

Fig 1 outlines the recruitment process and information on the interventions received. The study consisted of experimental (n = 16) and control arms (n = 8). The experimental arm comprised two separate groups (2-days and 1-day of NHE training) of eight participants and the control arm comprised just one group (no NHE training, n = 8). The twenty-four male soccer players (age [yrs] = 2-day group: 16.25[0.89], 1-day group: 16.63 [0.74], control: 16.50 [0.76]; height [cm] = 2-day group: 172.00 [6.95], 1-day group: 176.38 [5.76], control: 175.63 [4.69]; mass [kg] = 2-day group: 65.88 [9.06], 1-day group: 66.38 [4.60], control: 65.25 [5.68]) were all recruited from the same team. To allocate participants to the groups, and to ensure that those groups had as equal a proportion of players in each position as possible, players were categorised according to their playing position in the game (wide defender, central defenders, wide midfielders, central midfielders and strikers), and then randomly allocated into one of the three aforementioned groups. This randomisation procedure was justified on the basis of observed positional differences in sprint and jump performance; thus the confounding effect of playing position was controlled. The groups were allocated by another trainer and all of the researchers were blinded to this allocation. The sample size estimation was computed using G*Power software (version 3.1.9.6). We conducted an a *priori* sample size calculation for the outcome 10 m sprint time. We set a type I error rate of 0.05 and 80% statistical power. The estimated effect size of Cohen’s d = 0.78 is based on a similar study from Chaabene et al. [12] on the effects of NHE on physical fitness in youth athletes, over a similar time period. The analysis indicated that seven participants per group would represent a sufficient sample. To account for potential dropout, eight participants per group were recruited.

### Procedures

Players were asked to follow their regular diet on the day of the fitness assessments and to not consume any stimulant drinks. Tests were conducted between 10 o’clock and 12 o’clock in the morning on four days of the week (Saturday, Monday, Wednesday and Friday), with one day of recovery between each testing day. The average temperature on the testing days was between 13ºC and 17ºC whilst humidity was between 3% and 4%. The players wore their usual soccer footwear and performed the tests on the natural grass surface that they were accustomed to playing on. The warm-up for the tests was the FIFA11+ protocol [22], executed with minor changes. All players were fully acquainted with the utilised fitness tests being familiarised with them through their previous training activities. The order of fitness assessments was as follows: on day one, anthropometric measurements were undertaken and included height, body mass and body fat percentage. On day two, the 5-0-5 test was used to evaluate COD ability. On day three, the participants undertook the jump tests. On day four, 10 m and 40 m sprint speed were measured. The rest interval between each effort in each of the various tests was three to five minutes.

#### Anthropometry

Stature and body mass were assessed between 8 am and 10 am on the first day of testing. The assessments were made by the same observer. Stature was assessed using a stadiometer (Seca 217 Stable stadiometer, Hamburg, Germany) and body mass was measured using an accompanying scales. Body fat was assessed using Harpenden skinfold calipers, (Burgess Hill, United Kingdom) following the protocol of Jackson and Pollock [23]. The body fat percentage was assessed using a seven-point formula as follows: 1-pectoral, 2-midaxillary, 3-triceps, 4-subscapular, 5-abdominal, 6-suprailiac, and 7-thigh. All the measures were made by the same observer on the right side of the body.

#### Horizontal jump

The standing long jump (SLJ) was employed to measure horizontal jumping performance and followed the protocol of previous researchers (ICC = 0.94) [24]. Participants were guided through an initial familiarisation trial during which the key aspects of execution were communicated to them. The participants stood behind a line marked on the ground with feet slightly apart and were asked to maintain a parallel foot position during both take-off and landing of the jump. The jump was measured using a long jump mat (Jump length pairs, Tanazma, Iran), marked in centimeters, with jump distance recorded corresponding to the position of the heels to the nearest point of contact upon landing. Each participant was permitted two trials with the best performance being used for further data analysis.

#### Sprint test

To measure sprinting speed, electronic timing gates were used (Newtest Powertimer 300-series testing system, Finland). This test has been shown to be highly reliable in the measurement of linear sprint speed (ICC = 0.89–0.9) in soccer players of a similar age [25, 26]. Distances of 10 m and 40 m were used to determine the sprinting speed of the players. Participants started in a split-legged stance with the preferred foot positioned 70 cm behind the first pair of photocells that marked the starting line. Four pairs of photocells were used (starting line, 10 m and 40 m). The photocells were positioned at hip height to enable the capturing of trunk movement, rather than a false trigger from a limb. Players performed two trials with the fastest used for further data analysis.

#### Change-of-direction test

The 5-0-5 test protocol was employed to measure the COD speed of the players. Electronic timing gates were used (Newtest Powertimer 300-series testing system, Finland) and the test has been shown to be highly reliable in the measurement of COD ability (ICC = 0.93) in soccer players of a similar age [26]. From a split-stance starting position, the participants were required to sprint 5 m before touching their foot on the demarcated line and then performing a 180 degree turn, positioning their body to sprint 5 m back through the start point. The players were allowed to use their preferred leg for braking and turning, however, they were asked to use the same leg for each effort. The photocells were positioned at hip height to enable the capturing of trunk movement rather than a false trigger from a limb. Players performed two trials with the fastest used for further data analysis.

### Exercise intervention

The duration of the intervention period was eight weeks and it took place during the pre-season period. One week before the start of the intervention, players’ workloads were regulated to prevent fatigue. During the eight-week intervention (Table 1), the 2-day group conducted NHE training on two days of the week before soccer training. The 1-day group executed NHE training on one day of the week (Tuesday) before soccer training and completed general skills (submaximal pass-and-move drills) training when the 2-day group was involved in its second NHE session. Completely separately, the control group performed submaximal pass-and-move drills during all NHE training segments. All training occurred between 10 and 12 o’clock in the morning on each day. Prior to each session, the players undertook a FIFA11+ style warm-up program [22], executed with minor changes. Each group then practiced according to their own NHE or control protocol as described above. Though it was impossible to blind the control group to the activities of the experimental group, to prevent group contamination, coaches closely supervised each protocol. The main (soccer) part of each training session was shared between all groups and was performed immediately after the implementation of the NHE and control protocols. All groups performed six soccer training sessions per week with the NHE protocol performed only on Tuesdays and Fridays only. During the pre-season, most training sessions included submaximal and maximal aerobic exercise (65–95% of maximum heart rate), small-sided games, interval training and very basic additional strength training. This strength training was delivered in a circuit format and was identical for all players (including the control group): bodyweight squats (12 repetitions), push up (15 repetitions), flat plank (20 s), step-up (10 repetitions), burpees (7 repetitions), side planks (20 s) and bicycle crunches (25 repetitions). The break between each station was 60 seconds and each player executed three circuits.

**Table 1:** Training load during the 2-day, 1-day and control group.

Group	Day 1 (sets x repetitions)			Day 2 (sets x repetitions)		
	2-day group	1-day group	Control	2-day group	1-day group	Control
Week 1	2x5	4x5	Skills	2x5	Skills	Skills
Week 2	2x5	4x5	Skills	2x5	Skills	Skills
Week 3	2x6	4x6	Skills	2x6	Skills	Skills
Week 4	2x6	4x6	Skills	2x6	Skills	Skills
Week 5	3x6	6x6	Skills	3x6	Skills	Skills
Week 6	3x6	6x6	Skills	3x6	Skills	Skills
Week 7	3x8	6x8	Skills	3x8	Skills	Skills
Week 8	3x8	6x8	Skills	3x8	Skills	Skills

Intensity: exercises were performed with maximal effort (intensity: 100%), Rest: 1–2 min between sets

One hour after each session that included their only NHE exercises of the week, the 1-day group provided an RPE as an estimate of workload. Similarly, one hour after the session that included their second bout of NHE exercises of the week, the 2-day group also submitted an RPE. The CR-10 Borg scale [27] was used only in training sessions in which the NHE was executed. Participants were asked to rate their level of exertion on the NHE only, focusing on the overall sensation of the activity and taking into account the feeling of physical stress during performance.

#### Execution of the Nordic hamstring exercise

The NHE was performed as per one of our previous studies in youth soccer players [6]. The NHE part of each session ranged from around 10 minutes in week 1 to 20 minutes in week 8 of the intervention. For performance of the NHE, participants were instructed to kneel on the ground with their ankles secured in place by a partner who orientated their weight downwards to prevent any movement of that joint. The participant who was executing the NHE was positioned such that the ankles were perpendicular to the lower leg. To execute the exercise, the participant gradually lowered the upper body, resisting the downward movement by contracting the hamstring and gluteal muscles and aligning the trunk and hips in a neutral position throughout performance. The arms were held around their chest and flexed at the elbow joints such that the palms of the hands were facing the shoulder joints. Participants were allowed to use their arms in the final stages of the movement to buffer the fall as their upper body approached the ground. From there, they ascended back to the start position using their arms before repeating to complete the assigned number of repetitions. Following completion of this, the participant swapped roles with their partner to repeat the process. This facilitated an inter-set rest of at least one minute [28].

### Statistical analyses

Statistical analyses were carried out using JASP (version 10.2, University of Amsterdam). The normality and equality of variances for all data were checked with the Shapiro-Wilk and Levene tests respectively. The independent samples t-test was used to compare the groups’ fitness test scores at baseline, and also to assess post-intervention mean RPEs. The repeated-measures ANOVA was used to detect statistically significant (p<0.05) changes in the dependent variables with Tukey-adjusted post-hoc tests conducted to identify statistically significant comparisons. Cohen’s *d* effect sizes (ES) were also computed and were classified as ‘trivial’ (<0.2) ‘small’ (>0.2–0.59), ‘moderate’ (>0.6–1.19), ‘large’ (>1.2–1.99), or ‘very large’ (>2) [29].

## Results

### Effect of the intervention: experimental group vs. control group

Both groups achieved a 100% attendance rate. There were no significant differences between the groups in any of the measured variables at baseline (p = 0.5–0.97). After the intervention, our analyses revealed significant group by time interactions for 10 m sprint (df = 1, F = 17.718, p<0.001, η^2^ = 0.120, d = 2.05 [0.57 to 3.53]), 40 m sprint (df = 1, F = 13.167, p = 0.001, η^2^ = 0.041, d = 1.09 [-0.23 to 2.4]) and COD (df = 1, F = 11.980, p = 0.002, η^2^ = 0.063, d = 1.25 [-0.09 to 2.59). There was no significant group x time interaction for SLJ. In the *post hoc* analyses, the experimental group demonstrated ‘moderate’ to ‘very large’, statistically significant, effect sizes in all measured performance variables, with the exception of the SLJ. By comparison, the control group experienced only small to moderate changes across all measured performance variables, only one of which was statistically significant. These findings are summarised in Table 2.

**Table 2:** Results of the experimental vs control analysis.

	Group	Baseline (Mean, SD)		Follow up (Mean, SD)		Change	% change	d	95% Confidence interval	Tukey (p<0.05)
10 m sprint (s)	Control	1.88	0.03	1.84	0.04	0.04	2.1	0.84 (moderate)	-0.39 to 2.06	0.23
	Experimental	1.89	0.04	1.75	0.06	0.14	7.4	3.02 (very large)	1.50 to 4.54	< .001
40 m sprint (s)	Control	5.70	0.07	5.64	0.06	0.06	1.1	0.89 (moderate)	0.12 to 1.66	0.006
	Experimental	5.70	0.09	5.56	0.06	0.14	2.5	1.94 (large)	0.98 to 2.91	< .001
Change of direction (s)	Control	4.11	0.09	4.02	0.12	0.10	2.2	0.58 (small)	-0.29 to 1.45	0.24
	Experimental	4.12	0.13	3.81	0.24	0.31	7.5	1.84 (large)	0.85 to 2.83	< .001
Standing long jump (cm)	Control	210.3	18.3	223.6	15.4	13.4	6.3	0.73 (moderate)	0.43 to 1.89	0.30
	Experimental	217.2	25.4	229.5	8.8	12.3	5.7	0.67 (moderate)	0.18 to 1.52	0.12

### Intervention comparison: 1-day group vs. 2-day group

There were no significant differences between the 1-day and 2-day training groups, following the intervention period, in any of the measured performance variables. In the *post hoc* analyses, both groups experienced significant increases in physical performance in all measured variables except the SLJ. With the exception of the SLJ, effect sizes for all measured variables were in the ‘large’ to ‘very large’ range. However, in three of the four measured variables, the 2-day group demonstrated larger effect sizes for increases in physical performance. These findings variables, the 2-day group demonstrated larger effect sizes for increases in physical performance. These findings are summarised in Table 3. Fatigue, as measured by RPE was significantly lower in the 2-day group (5.97 ± 0.17 AU vs. 4.91 ± 0.40 AU, p < .001) with a ‘very large’ effect size (d = 3.46 [1.83 to 5.04]).

**Table 3:** Results of the 1-day vs 2-day analysis.

	Group	Baseline (Mean, SD)		Follow up (Mean, SD)		Change	% change	d	95% Confidence interval	Tukey (p<0.05)
10 m sprint (s)	1-day group	1.88	0.04	1.72	0.07	0.04	8.5	3.10 (very large)	0.94 to 5.25	< .001
	2-day group	1.90	0.03	1.77	0.04	0.14	6.8	2.59 (very large)	0.64 to 4.54	< .001
40 m sprint (s)	1-day group	5.68	0.10	5.56	0.08	0.06	2.1	1.63 (large)	0.45 to 2.81	< .001
	2-day group	5.71	0.07	5.56	0.04	0.14	2.6	1.94 (large)	0.62 to 3.26	< .001
Change of direction (s)	1-day group	4.12	0.12	3.88	0.23	0.10	5.8	1.30 (large)	0.21 to 2.38	0.002
	2-day group	4.11	0.14	3.74	0.24	0.31	9.0	1.96 (large)	0.60 to 3.32	< .001
Standing long jump (cm)	1-day group	222.9	26.8	227.6	9.2	13.4	2.1	0.25 (small)	-1.03 to 1.53	0.943
	2-day group	211.5	24.3	231.4	8.5	12.3	9.4	1.04 (moderate)	0.35 to 2.43	0.138

## Discussion

The results of this study demonstrate that the addition of the NHE to a conventional programme of physical development in youth soccer players can result in substantial increases in sprint and COD speed, though not necessarily in jumping distance. Furthermore, though the execution of the volume-equated NHE seems to be highly effective in both 1-day and 2-day formats, there could exist a slight advantage if coaches prescribe the latter format with more efficient workload management a potential mechanism. These results show increases in performance in most of the fitness tests used in this study, such as the 10 m and 40 m sprint and COD tests, and thus add to the growing body of literature relating to the efficacy of the NHE in youth.

The increases in performance we report may owe to the combined effects of greater eccentric muscle strength and increased fascicle length [30] which can result in improvements in sprinting [31]. When executing the NHE, the bicep femoris muscle lengthens as hip and knee extension occurs [32] thus mimicking the lengthening action of the hamstrings during sprint activity. As sprinting speed increases, the bicep femoris muscle undergoes an increase in musculotendon stretch [33] resulting in structural changes such as the increased fascicle length that occurs after NHE training in male youth athletes [2]. This can have a positive effect on sprinting performance. Furthermore, strength adaptations can be denoted by increases in maximal eccentric contraction force and enhanced activation of the biceps femoris and semitendinosus muscles [34]. Currently, it is difficult to determine what combination of the above described factors could have contributed to performance enhancement but future work can focus on the relative contributions of these mechanisms.

A previous NHE intervention in young active males [34], of similar duration to the current study, revealed that increases in eccentric muscle strength were underpinned by changes in maximal voluntary contraction of the hamstrings, increased decelerative control of the trunk and enhanced electromyographic activity of the biceps femoris and semitendinosus muscles. Participants in that study executed 340 repetitions over the course of the six-week intervention period, resulting in a 15% increase in hamstring strength. Similarly, in a study like our own, Medeiros et al. [11] observed increases in hamstring strength of around 11% in a group of adult soccer players that executed approximately 472 total repetitions of the NHE, in a twice per week format, for eight weeks. The authors reported increases in fascicle length of around 9% in their participants; however, they intimated that less experienced populations may benefit to a greater extent with increases of up to 22% previously reported [35]. Previously, it was found that the execution of just 162 repetitions, in a six week NHE programme, resulted in 19% and 10% increases in relative peak force of the hamstrings in pre- and mid-pubertal soccer players, aged 11 and 14 years, respectively [6].

Though the above mentioned findings are encouraging, they relate specifically to the strength of the hamstrings so may not be entirely applicable to our own results; however, both Hammami et al. [36] and Freeman et al. [37] prescribed NHE programmes of 184 repetitions over six weeks and 110 over four weeks respectively, in youth athletes, examining their effects on various measures of running speed. Interestingly, Hammami et al. [36] reported increases in running speed of up to 5.74%, yet Freeman et al. [37] did not report any increases in speed over either 10 m or 40 m distances. Those authors suggested that the lack of improvement may have been due to what they classified as a “slow, non-specific eccentric contraction”. However, contrary to their results, we observed appreciable increases in both accelerative and maximum speed over the eight-week study period. Despite reporting enhanced eccentric hamstring strength, it could be that the total load of the prescribed NHE applied by Freeman et al. [37] was not sufficient to elicit an increase in sprint speed over the four week study period and so youth athletes may benefit from a higher volume of training over a longer period of time. In our study, the participants undertook 256 repetitions which resulted in ‘large’ to ‘very large’ effect sizes in all but one performance variable. This has potential implications for the transfer of training adaptations in response to the NHE and the likelihood that these can transfer to sport-specific skills such as sprinting and jumping.

In terms of periodisation, short term studies [38, 39], of just three week’s duration, have demonstrated the efficacy of dividing volume-equated training loads into a greater number of training sessions. Hakkinen and Kallinen [38] reported increases in isometric strength, muscle hypertrophy and activation when training was divided into two daily sessions, as compared to one, across two separate three week training periods. Similar results were observed by Hartman et al. [39] who reported larger increases in both strength and muscle activation when young male weightlifters trained twice daily, with similar volumes, as opposed to once. The authors suggested that despite there being no clear benefit to dividing training across days, an increase in isometric strength and electromyographic activity could potentially reduce the risk of sustaining injury. The lack of any statistically significant differences between the 1-day and 2-day groups in our study could be suggestive of a similar trend. Indeed, coaches should be able to use these programming formats interchangeably. However, the magnitude of the effect sizes in the respective groups suggest that there may be a practically important advantage to using the 2-day format. On this, previous research [40] has demonstrated reductions in maximal eccentric torque of up to 17% after a single set of the NHE. Despite undergoing equalised training loads, our 2-day group executed no more than three sets per session but our 1-day group executed as many as six. It is therefore possible that RPEs were higher in the 1-day group due to a greater intra-session volume of NHE. As the intensity of training can be directly inverse to its duration, dividing sessions could facilitate a lower level of exertion and greater programing efficiency [39]. This is reflected in our finding which shows consistently higher RPEs in the 1-day group in our study. Indeed, Ochi et al. [19] reported similar results in two groups of college students who undertook eleven weeks of volume-equated knee extension exercise training in 1- and 3-session per week formats. With both groups carrying out 72 repetitions per week, the researchers found that RPEs were higher in the one-session group than they were in the three-session group. This suggests that spreading the same amount of training across a higher number of sessions might regulate the negative effects of exertion on performance.

This research has several limitations. We were unable to include a measure of eccentric strength in this study and future investigations may add further important information if this can be achieved. Moreover, we did not include any measures of architectural changes to the muscle meaning conclusions on certain underpinning mechanisms would be speculative. Furthermore, we were unable to evaluate the biological maturational status of the participants while the specificity of the sample may not allow us to extrapolate the results to other sports and sports contexts.

## Conclusion

Our results clearly demonstrate the advantages that doing a programme of NHE training can have on physical fitness in youth athletes with a beneficial effect on sprinting, jumping and the ability to change direction rapidly. The study also demonstrates that these benefits can be gained after a moderate amount of training executed over a relatively short timeframe making the NHE an excellent option for youth strength and conditioning coaches who are constrained by a lack of time and resources. Though we found that both 1-day and 2-day per week training configurations were highly effective in enhancing physical fitness in youth soccer players, the performance gains when training was divided across two days was slightly higher in three out of four tests, whilst RPE was lower. We encourage coaches to adopt the training configuration that fits with the schedule of the sport that their athletes play. In youth populations in particular, options for incorporating RT into practice can be limited by the availability of the necessary expertise and resources. The NHE represents a safe and effective option that is relatively easy to coach and learn, thus its use in programmes of physical development for youth is recommended.

## Supporting information

S1 Data (XLSX)

S2 Data (XLSX)

S3 Data (XLSX)
